# A Simple Method to Train the AI Diagnosis Model of Pulmonary Nodules

**DOI:** 10.1155/2020/2812874

**Published:** 2020-08-01

**Authors:** Zhehao He, Wang Lv, Jian Hu

**Affiliations:** Department of Thoracic Surgery, The First Affiliated Hospital, College of Medicine, Zhejiang University, China

## Abstract

**Background:**

The differential diagnosis of subcentimetre lung nodules with a diameter of less than 1 cm has always been one of the problems of imaging doctors and thoracic surgeons. We plan to create a deep learning model for the diagnosis of pulmonary nodules in a simple method.

**Methods:**

Image data and pathological diagnosis of patients come from the First Affiliated Hospital of Zhejiang University School of Medicine from October 1, 2016, to October 1, 2019. After data preprocessing and data augmentation, the training set is used to train the model. The test set is used to evaluate the trained model. At the same time, the clinician will also diagnose the test set.

**Results:**

A total of 2,295 images of 496 lung nodules and their corresponding pathological diagnosis were selected as a training set and test set. After data augmentation, the number of training set images reached 12,510 images, including 6,648 malignant nodular images and 5,862 benign nodular images. The area under the *P*-*R* curve of the trained model is 0.836 in the classification of malignant and benign nodules. The area under the ROC curve of the trained model is 0.896 (95% CI: 78.96%~100.18%), which is higher than that of three doctors. However, the *P* value is not less than 0.05.

**Conclusion:**

With the help of an automatic machine learning system, clinicians can create a deep learning pulmonary nodule pathology classification model without the help of deep learning experts. The diagnostic efficiency of this model is not inferior to that of the clinician.

## 1. Introduction

Malignant tumours are a type of malady that seriously threatens human life and health. In China, although the 5-year survival rate of malignant tumours is increasing year by year [1], the morbidity and mortality still increase every year [2]. Among them, lung cancer ranks first in the incidence of malignant tumours in China [1]. The results of the study show that screening low-dose spiral CT for people at high risk of lung cancer can significantly reduce lung cancer mortality [3]. However, the ensuing problem is that the detection rate of pulmonary nodules is increased. The differential diagnosis of subcentimetre lung nodules with a diameter of less than 1 cm has always been one of the problems of imaging doctors and thoracic surgeons [4].

In recent years, research and application of artificial intelligence based on deep learning are in full swing. In the field of medicine, the use of deep learning techniques for the diagnosis of imaging [5] and pathological [6] images is emerging. However, deep learning is a subject with a high threshold, and such research often requires the in-depth participation of deep learning engineers. In order to further reduce the threshold of deep learning, people of insight have proposed the concept of automatic machine learning (AutoML) [7]. AutoML can completely automate the creation of the entire deep learning process, reducing the knowledge of researchers in various fields using deep learning for research work.

This study intends to use Microsoft's Custom Vision [8] AutoML system to train the model by learning the thin-layer CT imaging data of the lung nodules and the corresponding pathological diagnosis. Use the test data set to test the diagnostic model and compare the diagnosis of the clinician. Use the results to evaluate the effectiveness of the model.

## 2. Materials and Method

### 2.1. Training Set and Test Set

Retrieve the pathological diagnosis database of surgical specimens from the Department of Pathology, the First Affiliated Hospital of Zhejiang University School of Medicine, from October 1, 2016, to October 1, 2019. In the database, screen out the pathological diagnosis with a higher ranking in the pathological results of pulmonary nodules. According to the patient data selected by the above diagnosis, the CT images of the patient in the hospital imaging system are retrieved one by one according to the patient's medical record number. The inclusion criteria include the following:

- Must be CT images of lungs within 30 days before surgery
- The CT image of the lungs should be a high-resolution horizontal sequence CT image (layer thickness 1.0~1.25 mm)
- There is no limit to the size of the lung nodule, but they need to be spherical or quasispherical, the boundaries can be recognized, and the surroundings are surrounded by inflatable lung tissue, without atelectasis
- There is only one lesion in the same lung lobe, or there are multiple lesions, but they are all removed, and the pathological results after surgery are the same

Download the patient's high-resolution CT image sequence (DICOM format) from the imaging system, and record the pathological diagnosis corresponding to the nodule. Randomly select 90% of all nodules as the training data set and 10% as the test data set.

### 2.2. Data Preprocessing

**Figure 1:**
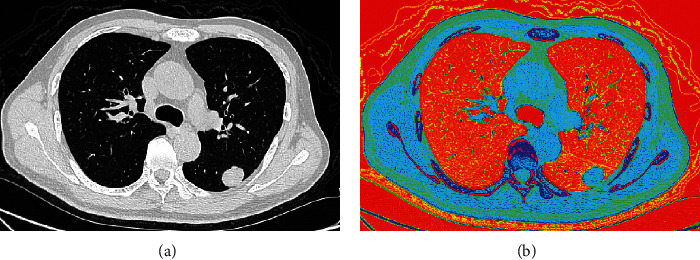
(a) A grayscale image of lung CT in a lung window and (b) a colour image after conversion.

Convert DICOM format images to bitmap images. The conversion scheme is as follows: in DICOM format, each pixel records the CT value whose unit is the Hounsfield unit. The range of the CT value is from -1000 to 1000. We specified for each CT value the only colour corresponding to it. Through this conversion, we get a colour CT bitmap image (Figure 1).

Select the images in the sequence that contain a lung nodule with the diameter of the lung nodule in the image not less than 80% of the largest diameter of the nodule. Crop the selected bitmap image to obtain an approximately square rectangular image containing the nodule image. The side length of the cropped image should be between 2 and 3 times the diameter of the nodule. Moreover, the nodule pattern is located approximately in the middle.

### 2.3. Data Augmentation

Perform the following operations on the training set image: rotate 90 degrees, 180 degrees, and 270 degrees clockwise, flip horizontally, and flip vertically. The above means make the training data set data increased by six times.

### 2.4. Training a Deep Learning Diagnostic Model

Visit https://www.customvision.ai, register a new account, and log in. Create a new training project, and select “Classification” for the “Project Type” option, “Multiclass (Single tag per image)” for the “Classification Types” option, and “General” for the “Domains” option. Upload all the training data set images, and add labels to the images according to the pathology type, and then start training. Wait for a moment, and record the training result data after the training is completed.

### 2.5. Evaluate the Trained Model with Test Data Set Images

Upload the test data set images on the test page to test the trained model. Since each nodule contains multiple test images, upload and test each image, record the percentage of each diagnosis possibility for each image, and average the multiple images. The diagnosis with the highest percentage is the final predicted diagnosis.

Invite three thoracic surgeons. View the lung nodules in the CT images corresponding to the test data set one by one, and diagnose according to the pathological grouping of the training data set. Make statistics after comparing the actual pathological results.

## 3. Results

Finally, a total of 2,295 images of 496 lung nodules and their corresponding pathological diagnosis were selected as a training set and test set. After data augmentation, the number of training set images expanded to 6 times before and eventually reached 12,510 images, including 6,648 malignant nodular images and 5,862 benign nodular images (Table 1).

**Figure 2:**
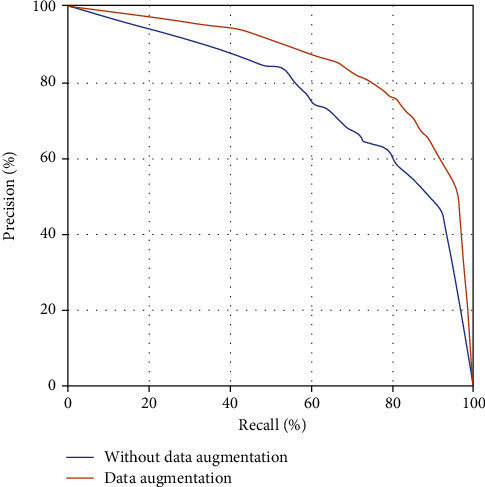
The *P*-*R* curve of the trained model with and without data augmentation.

The model trained using the training data set without data augmentation has a training result with a 50% probability threshold, the accuracy rate is 69.7%, the recall rate is 67.0%, and the area under the curve is 0.738. The training results of the model trained with the data augmentation training data set are as follows: at a 50% probability threshold, the accuracy rate is 78.8%, the recall rate is 76.2%, and the area under the curve is 0.836. After data augmentation, the area under the curve of the model is more excellent than before (Figure 2).

Use the model trained with enhanced data to make diagnostic predictions on the test data set. For benign and malignant classification, the model trained after data augmentation can reach a sensitivity of 88.24%, a specificity of 90.91%, and an overall accuracy rate of 90.0%. For pathological classification, the classification accuracy rate is 78%. For this test data set, three clinicians judged that the average sensitivity of benign and malignant classification is 86.27%, the average specificity is 65.66%, the average overall accuracy rate is 72.67%, and the average pathological accuracy rate is 48.67% (Table 2).

For the model trained after data augmentation and the three doctors, ROC curves are constructed for the diagnosis of benign and malignant nodules, which are used to judge their diagnostic value for the test data set. The area under the curve (AUC) corresponding to the model was 0.896 (95% CI: 78.96%~100.18%), and the area under the curve values corresponding to the three doctors were 0.759 (95% CI: 62.17%~89.70%), 0.775 (95% CI: 63.97%~90.93%), and 0.745 (95% CI: 60.12%~88.90%). The results mean that the model has a high value for the diagnosis of benign and malignant nodules in test data sets, and the corresponding optimal cutoff value is 0.791 (at this time, the sensitivity is 88.2% and the specificity is 90.9%). Moreover, the area under the curve is greater than that of the three doctors. However, the *P* value is not less than 0.05 (Table 3).

## 4. Discussion

Since deep learning has shown high accuracy in many computer vision tasks, in recent years, the research field of lung nodule detection and classification based on deep neural networks has rapidly heated up [9]. However, deep learning is a profession with a high threshold. Such research must rely on the participation of experienced deep learning engineers. In this study, the authors did not deeply study deep learning algorithms and specific operating practices. Only after a rough understanding of deep learning principles, an automatic deep learning system was used to create a deep learning diagnostic model. In this study, less than 500 cases of pulmonary nodules were collected as training data sets. Although the amount of data is not large, the final diagnostic model is still satisfactory and can be equivalent to the diagnosis of human doctors.

In previous studies [10], professional deep learning frameworks were often used to directly read lung nodule data in DICOM format to train models. However, in this study, Custom Vision can only read image data for training. To this end, we must convert DICOM format images into image format data for model training.

In the CT image of lungs in DICOM format, the data of each pixel is between -1000 and 1000. In other words, the CT machine can recognize 2000 different density differences in the human body. The CT values of human organs are mostly concentrated in a relatively narrow range. In order to facilitate display and doctor reading, DICOM format images will be displayed as grayscale images through different window width and window level values. The doctor can very sensitively perceive the difference in the CT value within the window width by reading the CT image with the naked eye. The disadvantage is that the CT value outside the window width will eventually be displayed as completely white or completely black. This image conversion will lose data.

In order to avoid losing data, we have created an image conversion method. CT can identify 2000 different gray levels in the human body. In the computer, taking the 24-bit colour bitmap as an example, the number of colours that can be displayed is 16,777,216. Therefore, each different CT value in the DICOM format can be given a corresponding colour, so that all the information in the DICOM format image can be completely retained. In the colour image after conversion, human eyes cannot recognize the slight difference between some colours. However, for computer processing, it has entirely different colours.

Lung adenocarcinoma is the most common type of pathology in non-small-cell lung cancer, and it accounts for about 50% of all lung cancer patients [11]. With the changes in the epidemiology of lung cancer, the International Association for the Study of Lung Cancer (IASLC), the American Thoracic Society (ATS), and the European Respiratory Society (ERS) formed a joint working group in 2011 to announce a new classification method for lung adenocarcinoma [12]: atypical adenomatous hyperplasia (AAH), adenocarcinoma in situ (AIS), minimally invasive adenocarcinoma (MIA), and invasive adenocarcinoma cancer (IAC). It is generally believed that AAH, AIS, MIA, and IAC are different stages of early non-small-cell lung cancer during the progression of the disease [13]. Statistical analysis of the prognosis of different types of lung adenocarcinoma revealed that AAH, AIS, and MIA have an excellent prognosis [14], and their 5-year survival rate can reach almost 100%. The 5-year survival rate of invasive adenocarcinoma is significantly lower than that of the previous three types.

In the choice of surgical procedures, for the types of AAH, AIS, and MIA, recent studies [15] have been more inclined to perform sublobar resection (pulmonary wedge resection, segmentectomy, and combined subsection resection). The survival rate and local recurrence rate are not significantly different from those of lobectomy. Some scholars [16] even believe that because of the types of AAH, AID, and MIA, the possibility of lymph node metastasis is extremely low. Stereotactic body radiotherapy (SBRT) treatment of these types of lesions can achieve similar treatment effect to traditional surgery. After the lesion reaches the level of invasive adenocarcinoma, lobectomy is more recommended.

In the process of rapid intraoperative pathological diagnosis, due to the influence of factors such as the material limitation, it is sometimes difficult for pathologists to distinguish between AAH, AIS, and MIA [17]. The three types of lesions have an excellent prognosis, and the clinical significance of surgical guidance is almost the same. Therefore, these three types are combined into a group as a low-risk group, and invasive adenocarcinoma is considered to belong to a high-risk group. Therefore, the classification model of deep learning can be more focused on identifying whether the lesion is invasive adenocarcinoma, which is of great significance for the formulation of surgical procedures and the prediction of disease prognosis.

In this study, the number of benign diseases is relatively small. For example, there are only 13 cases of pulmonary sclerosing hemangioma. If such a small number of cases are directly input into the deep learning engine for learning, it is bound to fail to obtain good results. Therefore, various forms of data augmentation are necessary. For image data, pure data augmentation methods generally include geometric transformation. In this study, the flip and rotation operations in geometric transformation are used. Rotating and flipping the image of a lung nodule do not affect the essence and characteristics of the image. This operation method is simple, but the effect is pronounced. The model uses the enhanced image for training, which increases the area under the curve by about 0.1 compared to the previous one.

The two most basic indicators in the fields of deep learning related to information retrieval, classification, recognition, translation, etc. are the recall rate and precision rate. Recall rate = true positive/(true positive + false negative), and precision rate = true positive/(true positive + false positive). Therefore, the recall rate is the sensitivity in medical diagnosis, but the precision rate is not specific. Nonetheless, the relationship between the precision rate and recall rate is similar to the relationship between sensitivity and specificity: precision and recall affect each other, and the ideal situation is, of course, both high precision and recall. But under normal circumstances, precision rate is inversely proportional to recall rate.

Therefore, similar to the ROC curve formed by the correlation between sensitivity and specificity, the relationship between the precision rate and recall rate can also build a *P*-*R* curve, where the recall rate value is used as the *x*-axis and the precision rate value is used as the *y*-axis to indicate the different relationship between precision and recall. The average precision rate represents the average value of the precision rate during the change of the recall rate from 0 to 1, that is, the integration of the precision rate during the shift in the recall rate from 0 to 1, which is equivalent to the area under the *PR*. The area surrounded by the *x*- and *y*-axes (area under the *P*-*R* curve). In this way, the comparison between multiple models becomes intuitive. You only need to place the *P*-*R* curves of various models in the same coordinate system and compare the area under the curve.

By analyzing the diagnosis results, the AUC value corresponding to the ROC curve of the neural network is 0.896, indicating that the model is of higher value for the diagnosis of benign and malignant nodules in test data sets. The model can achieve 90% accuracy for benign and malignant classification and 78% accuracy for pathological classification. Moreover, the AUC value is higher than that of the three doctors. However, the *P* value is not less than 0.05, indicating that the model's diagnostic efficiency of benign and malignant classification is similar to that of the clinician.

When clinicians diagnose lung nodules, the sensitivity is not much different from that of the diagnostic model, but the specificity is significantly lower than that of the diagnostic model. The possible reason is that as a clinician when diagnosing pulmonary nodules, they tend to increase sensitivity, increase the detection rate of potentially malignant tumours, and reduce the rate of missed diagnosis. As a result, the false positives are high and the specificity is reduced.

## 5. Conclusion

This study shows that with the help of an automatic machine learning system, clinicians can create a deep learning pulmonary nodule pathology classification model without the help of deep learning experts. The diagnostic efficiency of this model is not inferior to that of the clinician, but the deep learning algorithm model will not replace the status of clinicians and radiologists. On the contrary, it can effectively help clinicians and radiologists in clinical work.

## Figures and Tables

**Table 1:** Training set and test set.

Type		Nodules			Images		
Benign or malignant	Pathology	Training set	Test set	All	Training set	Test set	All

Malignant	AAH/AIS/MIA	131	6	137	400	18	418
	IAC	72	8	80	460	36	496
	Metastatic cancer	54	3	57	248	10	258
	All	257	17	274	1108	64	1172

Benign	Chronic inflammation/granuloma	91	16	107	556	92	648
	Intrapulmonary lymph nodes	42	11	53	119	28	147
	Hemangioma	12	1	13	77	2	79
	Hamartoma	44	5	49	225	24	249
	All	189	33	222	977	146	1123

All		446	50	496	2085	210	2295

**Table 2:** Diagnosis results of the trained model and the doctors on the test data set.

	Sensitivity (%)	Specificity (%)	Accuracy rate (%)	Pathological accuracy rate (%)
Trained model (data augmentation)	88.24	90.91	90	78
Doctor A	88.24	63.64	72	46
Doctor B	88.24	66.67	74	48
Doctor C	82.35	66.67	72	52
Doctor average	86.27	65.66	72.67	48.67

**Table 3:** AUC and ROC curve best cutoff of the trained model and the doctors.

	AUC	Optimal cutoff	Sensitivity (%)	Specificity (%)	*P* (compared to the trained model)
Trained model (data augmentation)	0.896	0.791	88.2	90.9	
Doctor A	0.759	0.519	88.2	63.6	0.1212
Doctor B	0.775	0.549	88.2	66.7	0.1673
Doctor C	0.745	0.490	82.4	66.7	0.0963

## Data Availability

The data and materials in the current study are available from the corresponding author on reasonable request.
